# Epidermoid cyst within an intrapancreatic accessory spleen exhibiting abrupt changes in serum carbohydrate antigen 19-9 level: a case report

**DOI:** 10.1186/s40792-020-00892-z

**Published:** 2020-06-12

**Authors:** Chisato Takagi, Nobuo Hoshi, Yutaro Kikuchi, Hirofumi Shirakawa, Moriaki Tomikawa, Iwao Ozawa, Shoichi Hishinuma, Yoshiro Ogata

**Affiliations:** 1 Department of Hepato-Biliary-Pancreatic Surgery, Tochigi Cancer Center, 4-9-13, Yohnan, Utsunomiya, Tochigi, 320-0834 Japan; 2 Department of Pathology, Tochigi Cancer Center, 4-9-13, Yohnan, Utsunomiya, Tochigi, 320-0834 Japan

**Keywords:** CA 19-9 antigen, Epidermoid cyst, Intrapancreatic accessory spleen

## Abstract

**Background:**

Epidermoid cyst within an intrapancreatic accessory spleen (ECIAS) is a rare disease. While the detection of solid components relevant to an accessory spleen is a key diagnostic finding, the differential diagnosis between ECIAS and malignant tumors is difficult without resection in patients with no other findings of an accessory spleen.

**Case presentation:**

A 73-year-old male was found to have an elevated carbohydrate antigen (CA) 19-9 level (95 U/mL) at an annual checkup, and a cystic lesion in the pancreatic tail was located by abdominal ultrasound. Abdominal magnetic resonance imaging (MRI) revealed a multicystic mass, 24 mm in diameter, which exhibited varying intensities on T2-weighted images. There were no findings suggesting solid components on contrast-enhanced computed tomography and magnetic resonance imaging. Re-evaluation of serum CA 19-9 level revealed a rapid increase to 901 U/mL, which declined to 213 U/mL 3 weeks later. Ruling out the lesion’s malignant potential was difficult, and the patient underwent distal pancreatectomy with splenectomy. Histological findings revealed an ECIAS including multiple cysts, with the mucinous component of each cyst exhibiting different stages of biological reaction; one ruptured cyst exhibited inflammatory changes.

**Conclusions:**

Careful observation for changes in serum CA 19-9 level and MRI findings might facilitate the diagnosis of ECIAS without a solid component by imaging studies.

## Background

Epidermoid cyst within an intrapancreatic accessory spleen (ECIAS) is a rare, benign disease that does not require resection; however, most patients with ECIAS undergo distal pancreatectomy because of challenges in the accurate diagnosis without histopathological examination. Several retrospective analyses of the clinical characteristics of ECIAS suggest that the detection of solid components relevant to an accessory spleen might be a key diagnostic finding [1, 2], whereas few cases are preoperatively diagnosed and closely observed based on such findings [3, 4]. However, some studies have reported ECIAS in patients without the findings of an accessory spleen [1, 5]. Additionally, patients with ECIAS might display high serum levels of carbohydrate antigen (CA) 19-9, suggesting malignancy [3, 5]. In these cases, it is challenging to distinguish ECIAS from malignancies including mucinous cystic neoplasms and intraductal papillary mucinous neoplasms [1, 2]. Therefore, accumulation of cases is warranted, especially in patients with ECIAS who do not display findings of solid components on radiological examinations. We herein report the case of a patient with an ECIAS accompanied with an abrupt increase in serum CA 19-9 level, which later declined to below the normal upper limit.

## Case presentation

**Fig. 1:**
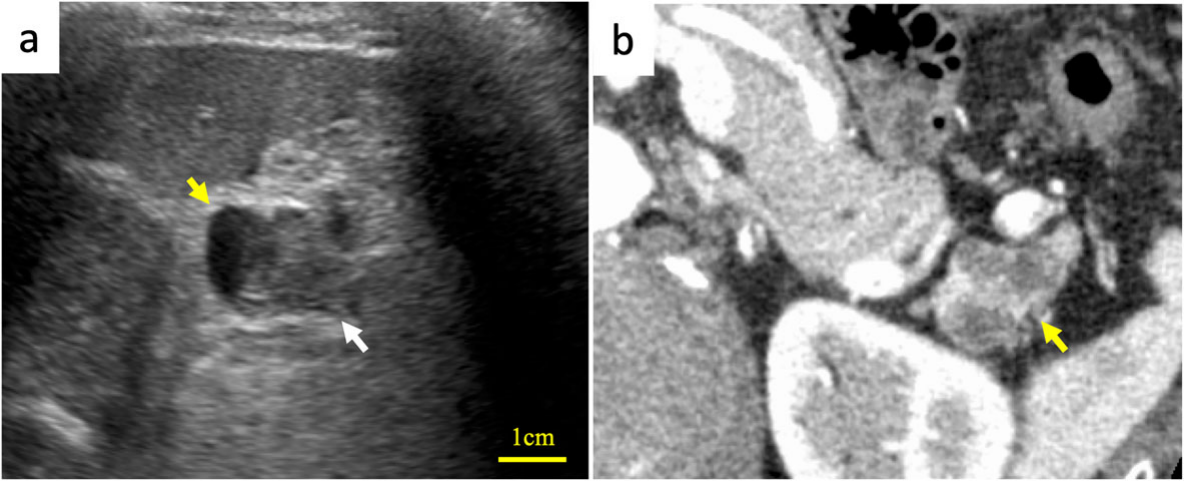
**a** Abdominal ultrasound showing a cystic lesion in the pancreatic tail (yellow arrow) and a slightly hyperechoic component (white arrow) in the cystic lesion. **b** Contrast-enhanced abdominal computed tomography shows a cystic mass with a diameter of 24 mm (yellow arrow)

**Fig. 2:**
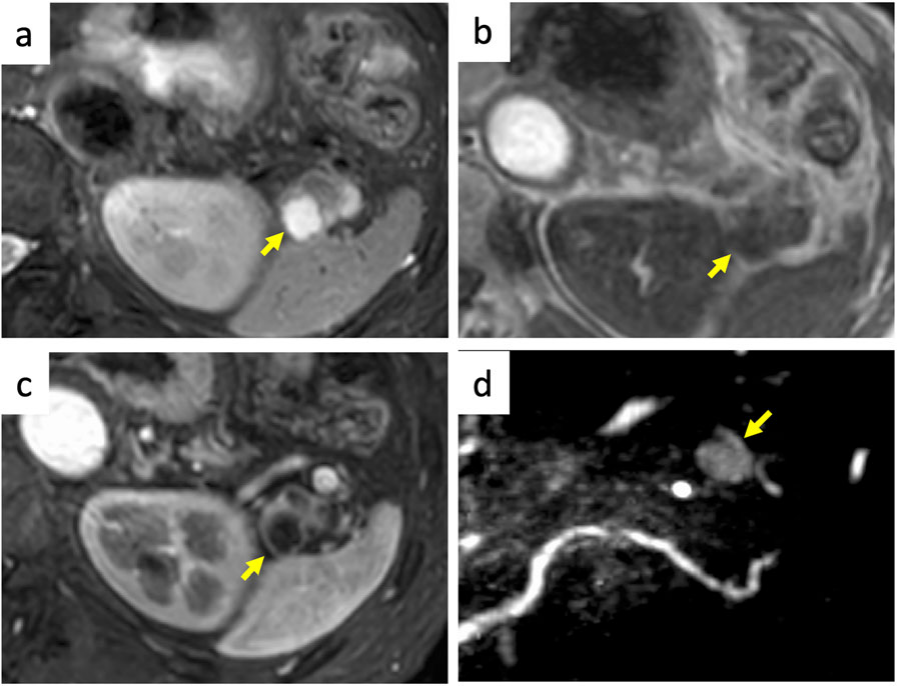
Magnetic resonance images. **a** Cystic components show varying intensities on T2-weighted images (yellow arrow). **b** Cystic components exhibit low intensity on T1-weighted images (yellow arrow). **c** Cystic wall is enhanced on contrast-enhanced magnetic resonance imaging (yellow arrow). **d** Magnetic resonance cholangiopancreatography showing that there is no indication to suggest a connection between the pancreatic lesion and the main pancreatic duct

A 73-year-old male, who had been undergoing annual blood tests and abdominal ultrasound for more than 10 years as part of health checkups, was found to have elevated CA 19-9 level (95 U/mL) and a cystic pancreatic lesion. The patient was asymptomatic and had an otherwise normal laboratory examination. Abdominal ultrasound revealed a cystic lesion in the pancreatic tail, and a slightly hyperechoic component was detected in the cystic lesion (Fig. 1a). Abdominal dynamic contrast-enhanced computed tomography (CT) revealed a multicystic mass, 24 mm in diameter, with no solid components observed in the entire lesion (Fig. 1b). Abdominal magnetic resonance imaging (MRI) revealed that the intensity varied across the cysts on T2-weighted images and that the intensity was generally low on T1-weighted images (Fig. 2a, b). The cystic capsules were enhanced but did not exhibit the same intensity as that of the spleen; furthermore, no solid components were detected in the cystic wall by gadolinium-ethoxybenzyl-diethylenetriamine pentaacetic acid contrast (Fig. 2c). On magnetic resonance cholangiopancreatography, the lesion exhibited high intensity and there were no findings of a connection between the lesion and the main pancreatic duct (Fig. 2d). Furthermore, on CT and MRI, there were no findings suggesting regional lymph node metastasis and distant metastasis related to the lesion. All potential malignancies except for those originating from the pancreas were ruled out by esophagogastroduodenoscopy, colonoscopy, and chest CT.

**Fig. 3:**
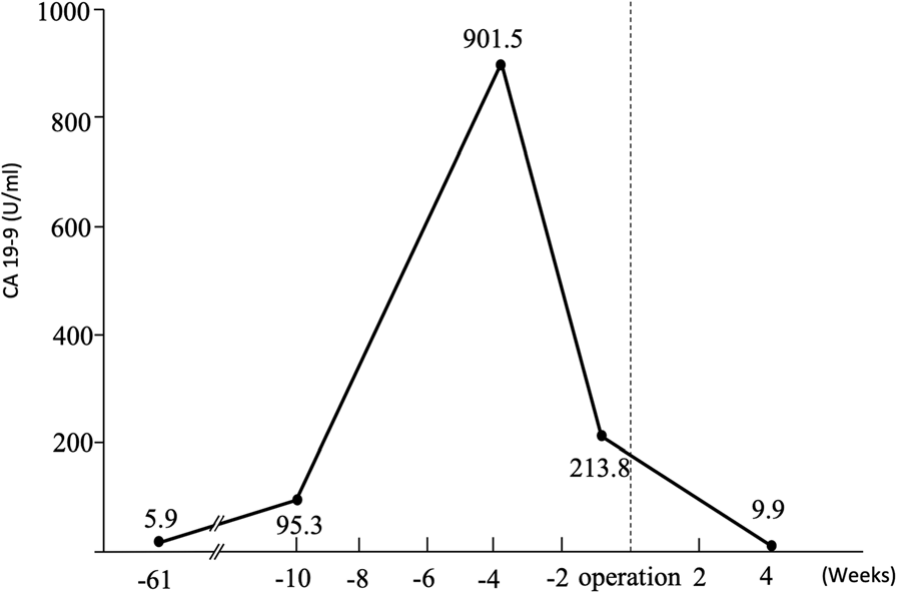
Changes in serum CA 19-9 levels over time

Re-evaluation of the patient’s serum CA 19-9 level after the completion of imaging studies revealed a rapid increase to 901 U/mL, which declined to 213 U/mL 3 weeks later (Fig. 3). Based on the difficulty in ruling out the lesion’s malignant potential, the patient underwent distal pancreatectomy with splenectomy. Intraoperative inspection and manipulation confirmed that the lesion was limited to the pancreas, and there were no findings of distant metastasis and regional lymph node metastasis.

**Fig. 4:**
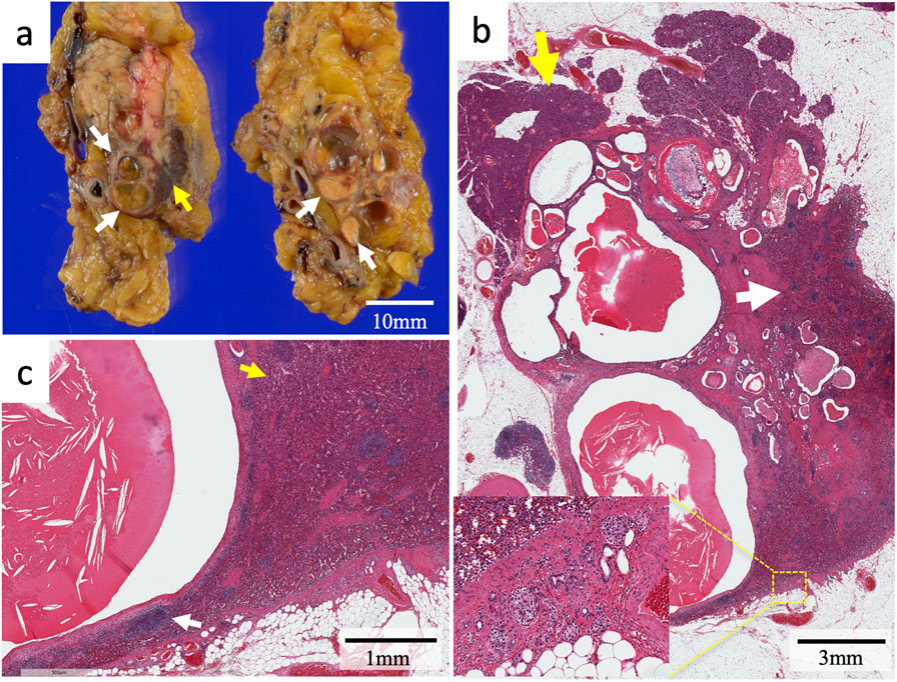
Macroscopic appearance and histological examination of the resected specimen. **a** Macroscopic appearance. Accessory spleen (yellow arrow) and multiple cystic lesions containing organized mucinous (white arrow) in the pancreatic tail. **b** A loupe image of the left side specimen in Fig. 4a. Pancreatic tissue (yellow arrow) and accessory spleen (white arrow) were detected on the upper side and the right side of the lesion, respectively. Inset: islets of Langerhans and pancreatic ducts were seen on the lower side of the lesion. **c** Accessory spleen consisted of red pulp (yellow arrow) and white pulp (white arrow) and was located between the cysts

**Fig. 5:**
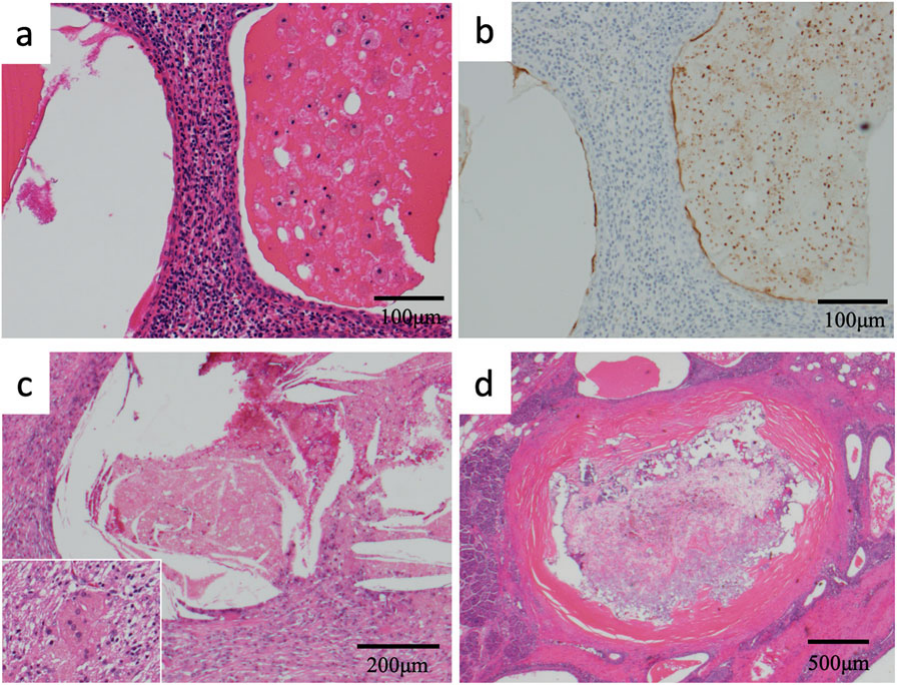
Histological findings of the cysts in the lesion. **a** Hematoxylin-eosin (HE) staining. Most cystic walls are lined by stratified squamous epithelium. **b** Immunopositivity for CA 19-9 is observed in the epithelial cells liming the cyst and in the cystic fluid. **c** HE staining. The cystic wall has lost stratified squamous epithelium. Mucin in the cyst contains cholesterol clefts. Inset: Foreign-body giant cells and fibroblasts are observed near the cystic wall. **d** HE staining showing the complete loss of epithelial cells. The cyst contains organized mucin inside, with a thick, hyalinized mucin outside

Macroscopic inspection of the resected specimen revealed that the entire lesion consisted of multiple cysts and accessory spleen (Fig. 4a). Histological examination showed that islets of Langerhans and pancreatic ducts were present around the lesion; therefore, the entire lesion was located within pancreatic parenchyma (Fig. 4b). The lesion comprised an accessory spleen and multiple cysts containing mucinous material. The accessory spleen comprised red pulp and white pulp, characteristic of a normal spleen, and was present outside the lesion and between the cysts (Fig. 4c). Most of the cystic walls were lined by stratified squamous epithelium (Fig. 5a). Regarding immunohistochemistry, both the epithelium and mucin depositions were positive for CA 19-9 (Fig. 5b). The mucinous material in the cysts exhibited different time courses of biological reaction. In one cyst, the cystic wall had lost stratified squamous epithelium, and foreign-body giant cells and fibroblasts were observed nearby the cystic wall (Fig. 5c). The organized mucin in the cyst contained cholesterol clefts. These findings suggested that this particular cyst had ruptured due to chronic inflammation. Another cyst comprised organized mucin with a thick, hyalinized mucin outside where the epithelial cells were completely lost (Fig. 5d). These findings suggested that this particular cyst had been undergoing chronic inflammation for a longer period of time than the aforementioned cyst.

Based on the operative and histopathological findings, the definitive diagnosis was ECIAS. The patient was discharged following an uneventful postoperative course. The CA 19-9 level was confirmed to have declined to below the upper normal limit 4 weeks after the resection.

## Discussions

As illustrated in the present case, patients with an ECIAS in the pancreatic tail might follow a characteristic course of an abrupt increase in CA 19-9 levels, followed by a decline; this feature, which is atypical for malignancies, provides support for the diagnosis of a benign disease. One retrospective analysis revealed that approximately 60% of reported ECIAS cases were associated with elevated CA 19-9 levels [1]. An elevated serum CA 19-9 level tends to raise the suspicion of a malignancy, which has led to surgery in reported patients with ECIAS. However, one study has reported that an elevated serum CA19-9 level is not a reliable marker in distinguishing benign lesions from malignant tumors in patients with cystic lesions in the pancreatic tail [6]. A decline in serum CA 19-9 levels, which is not a typical course in patients with malignancies, has not been reported in cases of ECIAS either. The mechanism underlying the characteristic changes in serum CA 19-9 level over time remains unclear. However, one study has proposed that the elevation of serum CA 19-9 level was associated with an increase in the intracystic pressure due to a CA 19-9-rich cystic fluid produced by the epithelium lining [3, 7–9]. Furthermore, cystic rupture is considered to be a mechanism of transport of intracystic tumor markers into circulation [9]. In the present case, we presumed that the elevation of serum CA 19-9 level was caused by an increase in the intracystic pressure that was accelerated by cystic rupture due to inflammation. The observed decrease in serum CA 19-9 levels might be explained by a reduction in the intracystic pressure following cystic rupture.

The present case also illustrates that the cystic components in ECIAS can exhibit different intensities on T2-weighted images on MRI. This finding, which has not been reported as a characteristic finding of ECIAS, can be plausible based on the histological findings of the present case. Specifically, the lesion comprised multiple cysts, including some with viscous mucin and others with organized mucin. Organized mucin in the cysts contained different properties including cholesterol clefts, macrophages, and fluid in the absence of cells and crystals. Similar findings in the cystic components have been previously reported in a patient with ECIAS [10]. Mainly, the properties of cystic components can range from low viscosity to high viscosity and solidification, reflecting the different stages of chronic inflammation. This finding, which implicates that the cystic components might arise at different timepoints during disease, can distinguish ECIAS from other diseases such as mucinous cystic neoplasms and intraductal papillary mucinous neoplasms.

The accurate diagnosis of ECIAS is challenging, especially in cases where there is no sign of a solid component that represents the accessory spleen by radiological evaluation. Solid components suggesting an accessory spleen on CT or MRI can be evaluated further by superparamagnetic iron oxide-enhanced MRI to confirm the accessory spleen [11]. However, some patients with ECIAS have no solid components. In a retrospective study, 20 of 52 patients and 14 of 28 patients with ECIAS had only cystic lesions by CT and MRI, respectively [1]. For proper diagnosis of ECIAS without resection, larger patient cohorts are warranted for the analysis of clinical characteristics of ECIAS without a solid component.

## Conclusions

ECIAS is a rare disease that poses a challenge for diagnosis without resection, especially in the absence of any findings suggesting the presence of an accessory spleen. However, the presence of multiple cystic lesions in the pancreatic tail exhibiting varying intensities on T2-weighted images should raise the suspicion of ECIAS. Although a high serum CA 19-9 level is observed in patients with ECIAS, we propose a close observation and re-evaluation of serum CA 19-9 levels after excluding malignancies associated with high serum CA 19-9 levels.

## Data Availability

The data supporting the conclusions of this article is included within the article.

## Abbreviations

ECIAS: Epidermoid cyst within an intrapancreatic accessory spleen
CA 19-9: Carbohydrate antigen 19-9
CT: Computed tomography
MRI: Magnetic resonance imaging
